# Follow-up of asthma control and quality of life after discontinuation of omalizumab in severe asthmatic patients

**DOI:** 10.1186/1939-4551-8-S1-A75

**Published:** 2015-04-08

**Authors:** Sibel Nayci, Eylem Ozgur, Cengiz Ozge

**Affiliations:** 1 Mersin University School of Medicine, Turkey

## Background

There is evidence that long-term omalizumab treatment might have disease-modifying activity, however, important questions concerning treatment duration after clinical improvement remain unanswered. This study aimed to evaluate follow-up of asthma control and quality of life after discontinuation of omalizumab in severe asthmatic patients previously treated with long term omalizumab.

## Methods

This is a prospective, observational study. Omalizumab therapy was stopped in 16 severe allergic asthmatic patients who previously treated with omalizumab over a 3 years period. Asthma Control Test (ACT), Asthma Quality of Life Questionnaire (AQLQ), pulmonary function test and severe exacerbations were recorded for one year at three month intervals after discontinuation of omalizumab.

## Results

The mean age was 53.5±9.5 and duration of asthma was 21.2±11.2 years. Serum total IgE level was 380.3±196 IU/mL. Mean duration of omalizumab treatment was 54.6±15 months. Loss of asthma control was documented in 10/16 patients (62.5%). The mean time to the first moderate to severe asthma exacerbation after discontinuation was 2.68±2.2 months. No correlation was found between time to loss of control and duration of omalizumab treatment. The mean score of ACT in the time of discontinuation of omalizumab decreased from 22.13±1.2 to 21.06±1.5 at 3th months (p=0.0001) and to 19.3 ±2.0 at 12th months of discontinuation (p=0.005). The number of exacerbation within the last 12 months increased from 1.3±0.9 to 3.4±3.2 (p=0.006), and the number of hospitalization increased from 0.12±0.26 to 0.6±0.9 within 12 months of discontinuation. The mean score of AQLQ decreased from 4.17±0.8 to 3.26±0.7 at 12th months of discontinuation (p=0.0001).

## Conclusions

The discontinuation of omalizumab after the successful long term therapy was associated of early loss of asthma control, moderate to severe exacerbation of the disease, and impaired quality of life. This suggest that the decision regarding discontinuation of omalizumab treatment should be undertaken individually after careful evaluation of benefits and risks in severe asthmatic patients eventhough they had been treated with a long enough course of omalizumab.

